# KET‐MCI: An open‐label trial of single‐dose ketamine treatment to improve depression in mild cognitive impairment

**DOI:** 10.1002/alz.095388

**Published:** 2025-01-09

**Authors:** Rachel Fremont, Amelia Karim, Adriana Feder, Georges Naasan, James Murrough, Julia Berman

**Affiliations:** ^1^ Icahn School of Medicine at Mount Sinai, New York, NY USA; ^2^ Icahn Mount Sinai School of Medicine, New York, NY USA

## Abstract

**Background:**

The U.S. Population is older today than it has ever been. It is estimated that 12‐18% of people over the age of 60 have mild cognitive impairment (MCI). 35‐85% of individuals with MCI have neuropsychiatric symptom with the prevalence of depression alone being between 25‐40%. Unfortunately, evidence suggests that traditional antidepressant medications may be less effective for individuals with MCI and depression (MCI‐D). Therefore, there is a need to develop novel effective treatments for depression in MCI.

**Method:**

The primary goal of this open‐label clinical trial is to explore whether a single sub‐anesthetic (0.5 mg/kg) dose of intravenous (IV) ketamine, an N‐methyl‐D‐aspartate (NMDA) receptor blocker that has been shown to have a rapid and strong anti‐depressant effect in individuals with major depressive disorder, is safe and tolerable in individuals with MCI‐D. Participants receive a single infusion of ketamine with follow up 24 hours, 48 hours, 72 hours, 1 week, and 1 month after infusion. Other study outcomes include the Montgomery‐Asberg Depression Rating Scale (MADRS), the mini‐mental status exam (MMSE), blood‐based biomarkers, and neuroimaging.

**Result:**

8 Individuals with MCI‐D have completed the single infusion, 5 have completed the study and 3 are in follow‐up. So far, no serious adverse events have been reported. Adverse events included transient hypertension during infusion, fall, anxiety, lightheadedness, brain fog, and headache. All participants completed the single infusion and no study participants have dropped out. 100% of participants responded to treatment, defined as 50% reduction in MADRS, at 24 hours (n = 8). Response rates at 1 week (n = 8) were 62% and at 1 month (n = 5) were 60%.

**Conclusion:**

Sub‐anesthetic IV ketamine appears to be safe and tolerable in individuals with MCI‐D and may improve depressive symptoms. Larger randomized controlled studies should be performed to evaluate the utility of this treatment in individuals with MCI‐D.

